# Protocol for a population-based Ankylosing Spondylitis (PAS) cohort in Wales

**DOI:** 10.1186/1471-2474-11-197

**Published:** 2010-09-01

**Authors:** Mark D Atkinson, Sinead Brophy, Stefan Siebert, Mike B Gravenor, Ceri Phillips, David V Ford, Kerina H Jones, Ronan A Lyons

**Affiliations:** 1 School of Medicine, Swansea University, Singleton Park, Swansea, SA2 8PP, UK; 2 School of Human and Health Sciences, Swansea University, Swansea, SA2 8PP, UK

## Abstract

**Background:**

To develop a population-based cohort of people with ankylosing spondylitis (AS) in Wales using (1) secondary care clinical datasets, (2) patient-derived questionnaire data and (3) routinely-collected information in order to examine disease history and the health economic cost of AS.

**Methods:**

This data model will include and link (1) secondary care clinician datasets (i.e. electronic patient notes from the rheumatologist) (2) patient completed questionnaires (giving information on disease activity, medication, function, quality of life, work limitations and health service utilisation) and (3) a broad range of routinely collected data (including; GP records, in-patient hospital admission data, emergency department data, laboratory/pathology data and social services databases). The protocol involves the use of a unique and powerful data linkage system which allows datasets to be interlinked and to complement each other.

**Discussion:**

This cohort can integrate patient supplied, primary and secondary care data into a unified data model. This can be used to study a range of issues such as; the true economic costs to the health care system and the patient, factors associated with the development of severe disease, long term adverse events of new and existing medication and to understand the disease history of this condition. It will benefit patients, clinicians and health care managers. This study forms a pilot project for the use of routine data/patient data linked cohorts for other chronic conditions.

## Background

Ankylosing Spondylitis (AS) is a chronic inflammatory arthritis affecting between 1 in 400 and 1 in 270 people [1,2]. AS is characterised by inflammation of the spine, resulting in progressive and irreversible fusion of the spine. Peripheral joints, particularly the hips [3] can also be involved, often requiring hip replacement surgery. In a significant number of patients, AS is also associated with inflammation of other organs such as the heart [4,5], eyes [6], bowel [7] and skin [8]. In common with most chronic inflammatory conditions, AS is heterogeneous, having a variable course and unpredictable episodes of exacerbation [9].

AS typically strikes people in their late teens or early adult life and runs for the remainder of the life-course. It therefore has a significant impact on employment and function and on the use of health and social care resources. Over 80% of patients report daily pain, 60% report daily use of drugs 20 years after diagnosis [10] and in a Dutch study only 54.2% of the AS cohort were participating in the labour work force [11].

Our study proposes to collect and link a range of complementary sets of data; including, robust clinical data from rheumatologists (diagnosis, MRI/radiograph images), existing routinely collected datasets such as the GP records, out-patients clinical data, in-patient activity, emergency department, laboratory/pathology data and social services databases and finally data collected directly from the patients themselves (disease activity, function, quality of life, work limitations).

Re-use of routine data is problematic and requires detailed knowledge of the datasets and their idiosyncracies. Information Governance challenges can also be a problem in such distributed datasets. These problems have been surmounted by the establishment of the Health Information Research Unit (HIRU) at the School of Medicine at Swansea University as part of the Welsh Assembly Government's commitment to the UK Clinical Research Collaboration (UKCRC). Its remit is to realise the potential of electronically held, person-based, routinely collected information to conduct and support health-related research. HIRU has set up the Secure Anonymised Information Linkage (SAIL) databank to bring together, link and anonymise the widest possible range of person-based data, and has done this using a split-file approach to anonymisation to overcome issues of confidentiality and disclosure in health-related data warehousing [12]. The SAIL databank operates within a robust series of guidelines in line with the Caldicott principles and the National Information Governance Board for Health and Social Care. [13].

The collection and linkage of data proposed here is unique and not currently feasible elsewhere. Due to the existing routine data linkages [12] and a new rheumatology network incorporating all rheumatologists in Wales, it is now possible to undertake a national AS cohort study identifying a well defined and characterised group, with the intention of expanding this strategy to other rheumatological conditions in the future.

## Methods/Design

### Aims

This study aims to develop a cohort of people with AS using existing data from clinical and routine sources and data collected from patient completed questionnaires.

### Recruitment and composition of the cohort

All patients living in Wales diagnosed with AS by a rheumatologist will be included. Examining all GP records in the Swansea area showed that there are 361 people registered with AS in 2006 out of 239,354 people registered with a GP. This gives a prevalence of 1.5 per 1000. Extrapolating this figure to the whole of Wales generates an estimated 4354 people in Wales with a diagnosis of AS. We plan to write to all patients with AS attending a rheumatologist in Wales as well as those registered with participating GPs, to ask for their consent to be included in this cohort. With this combined approach we anticipate a cohort of 2000 pseudonymised AS patients or 50% of all available AS patients.

### Data sources

The AS cohort will be built on the SAIL data bank [13] and will use SAIL's approach to data linkage and anonymity. In this approach, the data provider divides a data file into two parts; the personal information (name, date of birth, address, national health number) in the first part (file 1), and the clinical data in the second (file 2), with a joining key to link the two parts. The personal information (file 1) is sent to a third party (Health Solutions Wales) who match the information against a list of all people registered with the National Health Service in Wales. In cases where the NHS number is absent, a mixture of other identifiers is used for probabilistic matching. This creates a linking number (Anonymised Linking Field), which is then encrypted to become ALF_E. The other identifiers are deleted, giving File 3. File 3 is then sent to HIRU. File 2 is sent directly to HIRU by the data provider. Files 2 and 3 are then brought together at the SAIL databank where they are merged using the joining key, which is then deleted [12].

This growing databank already holds over a billion anonymised records from 13 databases and these can be anonymously linked at the individual record level [13]. The SAIL databank has been used in characterising latent autoimmune diabetes in adults [14], establishing the hospitalised prevalence of Crohn's disease and ulcerative colitis [15], and investigating differences in blood pressure measurements taken in primary and secondary care settings [16]. Linkage of routine data to trial cohorts has been done in relation to identifying patients for a depression trial [17], to facilitate recruitment for a diabetes trial [18], and to link environmental information about the domestic circumstances of pregnant women with routinely collected child health data [19].

Thus, the use of this data warehouse in a wide range of clinical and research settings using combinations of routinely collected and trial data has been established. With the triangulation of datasets (comparing the same data items from different datasets), the validity and reliability of single datasets can be assessed.

#### Clinical data from Rheumatology centres

All rheumatology centres in Wales are in the process of adopting the same electronic clinical database system: Welsh Arthritis and Rheumatology Dataset (WARD). This is implemented on the CELLMA platform (RioMed Ltd., Eastleigh, Hampshire, http://www.riomed.com). WARD will, as a minimum, record all rheumatological diagnoses, date of diagnoses and medications whenever the rheumatological teams see a patient, in addition to providing clinical data support during consultations. This system can be used to easily identify all patients with a specific rheumatological diagnosis (such as AS) across Wales. Historical information about existing patients will be entered into the WARD system.

In 3 participating centres, a pilot scheme will be carried out to examine the feasibility of using existing radiographs or MRI reports. The pilot will examine the numbers of images available and the ease of obtaining them. If the collection of radiographs and MRIs is feasible, the radiographs will be scored using both the Stoke system [20] for lumbar spine and the BASRI system [21], which includes hip involvement. The radiographs and reports will be requested and scored to be included in the rheumatology clinical dataset and the system will be rolled this out to the other centres.

#### Patient self-administered data (PAS cohort)

Many of the validated measures of disease severity in AS are patient-assessed indices, such as the Bath Ankylosing Spondylitis Disease Activity Index (BASDAI) [22] and the Bath Ankylosing Spondylitis Functional Index (BASFI) [23].

We have previously demonstrated that these patient self-administered severity questionnaires for AS can be delivered over the internet as well as by paper [24] and have used this system to conduct an internet-delivered RCT in AS [25]. We have adapted this methodology in the present study.

As a result of clinical pressures, these measures are currently not routinely collected by all rheumatologists. Therefore, all patients coming for an appointment with a rheumatologist will be asked to complete a paper-based questionnaire at the visit. As part of the process of consent to be included in the cohort, patients will be asked to consent to completing a questionnaire every 6 months, either online if they have internet access, or by post. The contents of the questionnaires are summarised in Table 1.

**Table 1:** Summary of questionnaires

Questionnaire	Time interval	Summary
Baseline	0	Co-morbidities, family history, age of diagnosis and first symptoms, BASDAI [22], BASFI [23], QOL [32], visits to professionals

Not at work	3 months	Previous occupation, activity impairment questionnaire

At work	3 months	Work limitations questionnaire (WLQ) [38], Work productivity and activity impairment questionnaire (WPAI-SHP) [39,40]

Follow up	6, 12 months	BASDAI [22], BASFI [23], QOL [32], other conditions

AS costs	9 months	Visits to professionals, tests done, other conditions, medications, costs of various aspects of treatment and disability

Exercise and fatigue	15 month	International Physical Activity Questionnaire (IPAQ) [33], BASDAI [22], BASFI [23], Behavioural regulation in exercise questionnaire (BREQ-2) [34], Pittsburgh Sleep Quality Index [35], Hospital Anxiety and depression Scale [36,37]

Medication	0,3,6,9,12,15 months	Medication

This data will be fed back into the clinical dataset (WARD) and will be available to the patients' rheumatologists to help inform the patients' ongoing clinical care. In addition, for those patients not registered with a rheumatologist in Wales, all consenting patients registered with the National Ankylosing Spondylitis Society (NASS) (http://www.nass.co.uk/) and those registered with participating GPs, will receive postal or internet versions of the questionnaire.

Questionnaire responses will be linked using the SAIL process.

A website has been developed to give access to the questionnaires (http://www.ashealth.co.uk/). It includes open access sections for news about the cohort project as well as AS issues in general. There is also a section giving factsheets about AS, a community page where members of the AS community can give information about themselves and a forum where members of the community can submit posts on any topic.

Within the site, there are pages restricted to people with AS who are willing to register. The site collects name and address data as well as the name of the participant's GP. This renders the resulting dataset linkable via the SAIL mechanism. GPs are written to and asked to confirm that the patients have AS.

#### Routine data sources

For the PAS cohort, the data collected on patients with a diagnosis of AS, as given by the rheumatologist, will be linked to other routinely collected datasets using the SAIL system. This linkage will allow us to follow the patient pathway through the NHS system both retrospectively and prospectively. Linkage with GP system data provides information about patients going back 10 years including; previous diagnosis, presenting symptoms, results of laboratory tests and previous medications. This dataset can be used to follow the patient at every visit to the GP and therefore record the development of associated conditions and co-medication. Linkage with in-patient data will record all hospital visits, surgery and treatment. Linkage with the mortality datasets will ensure the dataset remains relevant and can examine survival of included patients. Linkage with A&E datasets will give information on emergency visits. The linkage with the GP system can also identify patients who have a diagnosis of AS but are not currently seen by a rheumatologist in Wales. Before inclusion into the cohort, the GPs of these patients will be asked to confirm that AS has been diagnosed by a rheumatologist. This will allow us to gain a full picture of the spectrum of AS patients, including those living in Wales but seeing a specialist elsewhere and those whose AS is not severe enough to require specialist rheumatology follow-up. Therefore, the characterisation of AS patients, at all disease stages can be maximised by the routinely collected data held on them by the NHS and other public bodies using the existing SAIL databanks.

### Ethics

PAS has been granted ethical approval by the London Research Ethics Committee (08/H0718/64).

## Discussion

This cohort takes its participants from GPs (primary care practitioners) and rheumatologists. The study is supplemented by patient-supplied information in the form of a series of questionnaires. Patient consent is required to include patients in the cohort. In addition, consent is sought to link cohort participants to the existing databank of routinely collected data currently in the SAIL system. The ability to link cohort members anonymously to routinely collected data is one of the unique features of this cohort. GP data is widely used in research, and can give a wide range of information about treatment and associated conditions, but one must be aware of the various issues of data quality and completeness concerning primary care records [26]. Other sources of routine data such as in-patient and out-patient records can also be linked to the cohort and can help to enhance the data held on each patient.

The cohort will have immediate impact on giving an objective estimate of the cost of AS at each stage of the disease which will help inform the use of anti-TNF agents. This is of immediate importance to many patients who could benefit from these agents, once approved by NICE. Patients who potentially meet the NICE criteria for the anti-TNF agents could be easily identified as part of this cohort.

The existing routine data from the previous 10 years will enable retrospective cohort studies to identify early risk factors (such as early hip involvement [27]) for progression to severe AS (as defined by the need for surgery or disability benefit). These patients could then be selected for aggressive early treatment with anti-TNF agents.

The cohort can be used to rapidly identify potential patients for RCTs of anti-TNF agents and other new drugs. This cohort can be used to screen for specified inclusion criteria and estimate the numbers eligible for new trials in the area. This will facilitate recruitment as the individuals can be identified remotely and invited by their local rheumatologists or GP to participate in these studies. The process of screening and recruitment of participants to trials can then be significantly speeded up reducing the risk and cost to pharmaceutical companies, thereby leading to more efficient and cost-effective trials.

The collection of AS patients can inform the development of genetic work in this field and facilitate the development of potential vaccines and other targeted therapies as a follow on from this work. Therefore it will facilitate the UK participation within international consortiums on genetic research of inflammatory autoimmune conditions. Once established and phenotyped, it is intended that this cohort will be used for studies in conjunction with the rheumatology research networks established by the Arthritis Research Campaign (ARC). These future studies include the potential to link the data from this cohort with biological and genetic sample banks from consenting patients. There has been a long history of work looking at the genetics of AS in the UK. However, there is a bottle-neck in terms of identifying enough people to conduct repeat validation studies to confirm the importance of new regions of interest for susceptibility and severity genes. By far the largest study in AS genetics to date involved 1000 patients with AS and 1500 controls [28], while no genome-wide association study has yet been done in AS [29]. Historically, these studies have not tended to use patients from Wales for logistical reasons as it has previously been difficult to identify these patients. Therefore this well characterised cohort, together with the collection of newly diagnosed AS patients, would enhance the ability of researchers in the UK to conduct these validation studies. Linking genetic and biological samples with the routinely collected clinical data would also allow the investigation and identification of associations not possible by other means.

Phenotyping patients would include collecting measures that reflect the expression of the disease without describing the actual genetic make-up of the patient. In the case of AS, this would include; medically held data such as first symptoms, disease history and development, areas affected, co-disorders, severity, family history, age at onset, environmental data such as socio-economic status, physical activity levels, medications, inherent modifiers such as sex, ethnicity and finally biochemical/immunological measures from the laboratory such as markers for inflammation. Much of this data can be obtained from existing routine data and from the patient.

The cohort can be used to work with family members of AS patients in order to identify individuals at high risk of developing AS (such as the children of AS-affected women with a young age at onset [30]) to examine the early disease history of AS pre-diagnosis (using MRI scans) in order to examine interventions which may switch off or prevent AS.

This study therefore builds on and enhances existing UK resources and infrastructure. The methods used in this study are also relevant to other chronic conditions, and the PAS cohort is seen as a pilot for this strategy of phenotyping cohorts using the linkage of routine, clinical and patient-entered data.

This cohort will also allow easy collection of post-RCT and post-marketing surveillance data, which is crucial for new biologic agents whose long-term effects are unknown. The British Society for Rheumatology (BSR) is currently piloting measures and data collection for the development of a Disease Management Register for AS, including a biologics register [31] The PAS cohort could be used to directly contribute to and enhance this national register.

Thus, in conclusion the cohort can be used for a wide variety of research studies (burden/cost of disease, disease history, trial recruitment, genetic research, basic research of biological markers, and post trial surveillance). This is a pilot for undertaking the same method of cohort development in other chronic conditions.

## Competing interests

The authors declare that they have no competing interests.

## Authors' contributions

The protocol was conceived and designed by SB, SS, DVF and RAL with input from MBG, CP and KJ regarding analysis and data for collection. The first draft of the paper was written by MDA and subsequent drafts were amended and finally approved by all the authors.

## Pre-publication history

The pre-publication history for this paper can be accessed here:

http://www.biomedcentral.com/1471-2474/11/197/prepub
